# Bi-Mamba-Load: A bidirectional structured state space model with sequence relationship adaptive perception for electricity load forecasting

**DOI:** 10.1371/journal.pone.0355805

**Published:** 2026-11-02

**Authors:** Junwei Zhu, Lingzhi Xi, Chongyang Liao, Baoqiang Zhong, Jianxu Zhong, Lei Zhang

**Affiliations:** Information and Communication Branch, Southern Power Grid Energy Storage Co, Guangzhou, Guangdong, China; Chunghwa Telecom Co. Ltd., TAIWAN

## Abstract

The accurate prediction of electricity load is a critical component for smart-grid management, energy allocation, and grid stability. Traditional statistical methods and Convolutional Neural Networks (CNNs) often struggle with nonlinear relationships or long-range temporal dependencies, whereas Transformer architectures incur quadratic complexity in the sequence length. The Structured State Space Model Mamba provides linear sequence modeling but uses a unidirectional scan and a fixed treatment of multivariate dependencies. To address these limitations, we propose Bi-Mamba-Load, which combines a bidirectional Mamba encoder, a custom forget gate, and a Sequence Relationship Adaptive Perception (SRAP) module. Both encoder branches operate exclusively on the observed historical window, so no observations from the forecasting horizon are used as inputs. SRAP computes Spearman correlation statistics from the training split and performs dataset-level selection between channel-independent and channel-mixing tokenization; the selected route remains fixed for all samples in that dataset. Experiments across eight benchmark datasets indicate competitive or superior forecasting accuracy against the evaluated baselines while retaining linear scaling with the input length, subject to the constant-factor overhead of bidirectional scanning.

## Introduction

The transition towards smart grids has made the accurate prediction of electricity load a core underpinning for energy management and grid optimization [1–4]. Reliable long-term load forecasting (LTSF) not only enables power utility companies to optimize generator scheduling, reduce operational costs, and prevent blackouts but also provides essential data references for integrating intermittent renewable energy sources, thereby significantly enhancing the overall stability of the power system [5–8]. However, modern electricity networks generate massive volumes of multivariate time-series (MTS) data. These sequences are characterized by high volatility, non-stationarity, and complex inter-dependencies among different regional nodes or sensor variables, making precise forecasting a formidable challenge.

Historically, traditional statistical methods such as Autoregressive Integrated Moving Average (ARIMA) and Support Vector Regression (SVR) were widely employed for load forecasting. The principle of these methods lies in modeling linear trends or mapping data into high-dimensional spaces using kernel functions [9–12]. While highly interpretable, they rely heavily on manual feature engineering and exhibit poor adaptability when handling the highly non-linear and dynamic multivariate interaction effects present in modern smart grid data. With the advent of deep learning, Convolutional Neural Networks (CNNs) were introduced to automatically extract localized temporal features using sliding convolutional kernels [13–16]. However, the fundamental weakness of CNNs in this domain is their localized receptive field; they struggle to efficiently capture the long-range dependencies that dictate macroeconomic or seasonal electricity consumption trends. Subsequently, Transformer-based models revolutionized the field by utilizing self-attention mechanisms to establish global dependencies [17–19]. Despite their dominance, Transformers are intrinsically constrained by a quadratic computational complexity with respect to the sequence length, creating a severe computational bottleneck when processing the long-sequence historical data necessary for accurate long-term load forecasting [20–23].

Recently, Mamba, an architecture built upon Structured State Space Models (SSMs), has emerged as a promising solution for long-sequence modeling [24–28]. By introducing a hardware-aware selective scanning mechanism, Mamba achieves computational complexity that scales linearly with sequence length while preserving long-range dependencies. However, applying vanilla Mamba to electricity load forecasting reveals distinct limitations. First, its unidirectional processing does not allow an early token within the fully observed historical window to aggregate evidence from later observed tokens in that same window [29]. Second, its gating mechanism may prioritize proximal information, which can weaken the representation of early historical observations that remain relevant for long-horizon forecasting [30]. Finally, existing adaptations often enforce either channel-independent processing or channel mixing and therefore cannot select a tokenization route according to the training-set correlation structure of a particular dataset.

To address inefficient long-sequence processing and the modeling of multivariate dependencies, we propose Bi-Mamba-Load for electricity-load forecasting. The model uses a bidirectional encoder whose two branches process the same observed historical window in opposite orders, together with an improved Mamba+ block containing a forget gate designed to promote early-history retention. We also develop a Sequence Relationship Adaptive Perception (SRAP) module that computes the correlation structure from the training split and selects a fixed dataset-level route between channel-independent and channel-mixing tokenization.

The primary contributions of this work are summarized as follows:

- We introduce the Mamba architecture into the domain of electricity load forecasting, exploiting its linear computational complexity to efficiently process massive, long-sequence power grid data, presenting a robust alternative to attention-heavy models.
- We propose a novel bidirectional Mamba+ encoding architecture. By integrating a complementary forget gate and bidirectional scanning, the model breaks through the limitations of unidirectional SSMs in capturing periodic temporal dependencies and prevents the loss of early historical load patterns.
- We develop the SRAP module for multi-source heterogeneous data. By selecting a fixed dataset-level tokenization route from training-only Spearman correlation statistics, the module replaces manual dataset-specific choices between channel-independent and channel-mixing processing.

## Related work

This section systematically reviews the research trajectory of electricity load forecasting, transitioning from early statistical models to contemporary deep learning architectures, thereby providing a solid academic context for the proposed methodology.

### Traditional and early machine learning methods

Early approaches to electricity load forecasting primarily relied on statistical models such as ARIMA and Exponential Smoothing. While mathematically interpretable, they require strict stationarity assumptions and struggle with the highly volatile and non-linear nature of real-world grid data. To overcome this, machine learning algorithms were introduced to map complex features. For instance, Elattar et al. [31] utilized Support Vector Regression (SVR) combined with locally weighted techniques to model the complex non-linear relationships in local load demand, significantly outperforming traditional statistical methods. Recognizing the impact of external variables, Lahouar and Slama [32] proposed a Random Forest-based day-ahead load forecasting model that integrated expert input selection, effectively capturing uncertain external factors such as sudden temperature changes and holidays. More recently, ensemble methods have gained traction; Park et al. [33] designed an XGBoost-based model for very short-term load forecasting that incorporates day-ahead results to mitigate forecasting uncertainties caused by behind-the-meter photovoltaic (PV) generation. Despite these improvements, traditional machine learning models heavily depend on manual feature engineering, limiting their scalability across complex, multi-node smart grids.

### CNNs and RNNs-based forecasting models

With the proliferation of smart meter data, deep learning has become the dominant paradigm for load forecasting, significantly reducing the need for hand-crafted features. Recurrent Neural Networks (RNNs), particularly Long Short-Term Memory (LSTM), excel at sequence modeling. Kong et al. [34] proposed an LSTM-based framework specifically tailored for residential load forecasting, successfully tackling the high volatility and uncertainty inherent in individual household consumption behaviors. To capture local temporal features in parallel, Convolutional Neural Networks (CNNs) were widely adopted. Amarasinghe et al. [35] demonstrated that deep CNNs applied to historical energy sequences could effectively extract power consumption patterns at the individual building level. Furthermore, hybrid architectures have emerged to leverage the strengths of both paradigms. For example, Wang et al. [36] introduced a CNN-BiLSTM model equipped with an attention mechanism to address the strong volatility and complex multi-energy coupling in integrated regional energy systems. However, these architectures still face fundamental bottlenecks: LSTMs suffer from gradient vanishing over extended forecasting horizons, and CNNs are restricted by their localized receptive fields, failing to grasp global seasonal trends [37–39].

### Transformer-based forecasting models

To address the long-range dependency limitations of RNNs and CNNs, Transformer models based on self-attention have been extensively applied to the energy sector. Zhao et al. [40] introduced a Transformer-based day-ahead load forecasting model that effectively handles irregular and non-smooth historical power data, avoiding the local minima issues common in RNNs. Recognizing the hierarchical topology of power grids, Giacomazzi et al. [41] explored the Temporal Fusion Transformer (TFT) across different grid levels, achieving significant error reductions in week-ahead substation forecasting. Hybrid Transformers also serve as powerful feature extractors; Li et al. [42] designed a Transformer-LSTM hybrid model to accurately seize intricate dynamic relationships for industrial users, whose loads are notoriously random and volatile. Additionally, advanced variants have been developed to model spatial-temporal interactions, such as the Transformer Spatio-Temporal Graph Neural Network (TSTG) [43], which simultaneously captures temporal dependencies and nonlinear multi-energy interactions. Despite their predictive success, the *O*(*L*^2^) quadratic computational complexity of the self-attention mechanism renders Transformers highly resource-intensive when processing the massive, high-frequency multivariate sequences typical of modern power networks [44–46].

### State space models in time series

State Space Models (SSMs), particularly the recently proposed Mamba architecture, offer a compelling alternative by combining the parallel training efficiency of CNNs with the fast, bounded-memory inference of RNNs, achieving linear time complexity *O*(*L*). The application of Mamba to power system forecasting is an emerging frontier. Menati et al. [47,48] recently proposed PowerMamba, a deep state-space model that captures the latent dynamics of grid variables, achieving superior forecasting accuracy while drastically reducing the parameter count by 78% compared to leading Transformers. To handle complex multivariate characteristics, Feng et al. [49] developed DecMamba, which integrates series decomposition into the Mamba architecture to distinctively model high-frequency seasonal components and low-frequency trend components in multivariate load series. Furthermore, addressing the unidirectional scanning limitation of standard SSMs, Wang et al. [50,51] introduced Bi-TimeSSM, employing a bidirectional temporal encoding mechanism to capture complementary temporal patterns from both forward and backward directions.

Recent Mamba-based forecasting architectures provide particularly relevant comparisons. TimeMachine [52] combines four Mamba modules to model multiscale global and local contexts; MambaMixer [53] introduces selective mixing across both token and channel dimensions; and Bi-Mamba4TS [54] applies forward and backward Mamba+ scans together with a series-relation-aware tokenization decision. We therefore include these methods in the expanded experimental comparison.

**Summary and Motivation:** Transformers provide strong forecasting performance but incur quadratic self-attention complexity. Mamba offers a linear-complexity alternative, yet the direction of selective scanning, preservation of early historical evidence, and the treatment of cross-variable dependencies remain important design choices. Motivated by these issues, we combine dataset-level tokenization selection through SRAP with a bidirectional Mamba+ encoder and a gated residual path, and evaluate the contribution and computational cost of each component separately.

## Methods

### Problem formulation and preliminaries

We formulate the long-term multivariate electricity load forecasting task as follows. Given a multivariate historical load sequence $X_{in}=[x_{1},x_{2},...,x_{L}]\in {\mathbb{R}}^{L\times M}$, where *L* denotes the length of the historical look-back window and *M* represents the number of variables, including electricity load readings from different regional substations and related meteorological factors. Our objective is to predict the future load values over a horizon *H*, denoted as $X_{out}=[x_{L+1},x_{L+2},...,x_{L+H}]\in {\mathbb{R}}^{H\times M}$.

To efficiently process this long-sequence data, our framework builds upon the mathematical foundation of State Space Models (SSMs). A continuous-time SSM maps a 1-D input sequence x(t)∈ℝ to an output sequence y(t)∈ℝ via a hidden state $h(t)\in {\mathbb{R}}^{N}$. This dynamic system is formulated as a linear ordinary differential equation:


$$\begin{array}{c} h^{\prime }(t)=Ah(t)+Bx(t),\;y(t)=Ch(t) \end{array}$$ (1)

where $A\in {\mathbb{R}}^{N\times N}$ represents the state transition matrix, reflecting the internal system dynamics, while $B\in {\mathbb{R}}^{N\times 1}$ and $C\in {\mathbb{R}}^{1\times N}$ are projection matrices. For deep learning applications on discrete electricity data, the continuous parameters must be discretized using a predefined step size Δ. Employing the Zero-Order Hold (ZOH) rule, the discrete parameters are defined as:


$$\begin{array}{c} \overset{―}{A}=\exp(\Delta A),\;\overset{―}{B}=(\Delta A{)}^{-1}(\exp(\Delta A)-I)\cdot \Delta B \end{array}$$ (2)

This discrete formulation permits the model to be computed efficiently as a global convolution for parallel training. However, standard SSMs utilize time-invariant parameters, limiting their context-awareness. The Mamba architecture resolves this bottleneck by introducing a selective scanning mechanism, transforming the parameters *B*, *C*, and Δ into data-dependent functions [24]. This critical enhancement enables the model to selectively filter relevant information, including sudden load spikes and anomalous consumption patterns, while dynamically updating the hidden state. Consequently, Mamba maintains a linear computational complexity of *O*(*L*), establishing a highly efficient paradigm for long-sequence power grid forecasting.

### Overview of the proposed architecture

The overall architecture of the proposed Bi-Mamba-Load is illustrated in Fig 1. Unlike traditional models that employ rigid processing pipelines, our framework is designed to be highly adaptable to the diverse and complex spatio-temporal dynamics inherent in modern power grids. The architecture seamlessly integrates data normalization, adaptive tokenization, and bidirectional state space modeling into a cohesive end-to-end forecasting pipeline.

**Fig 1:**
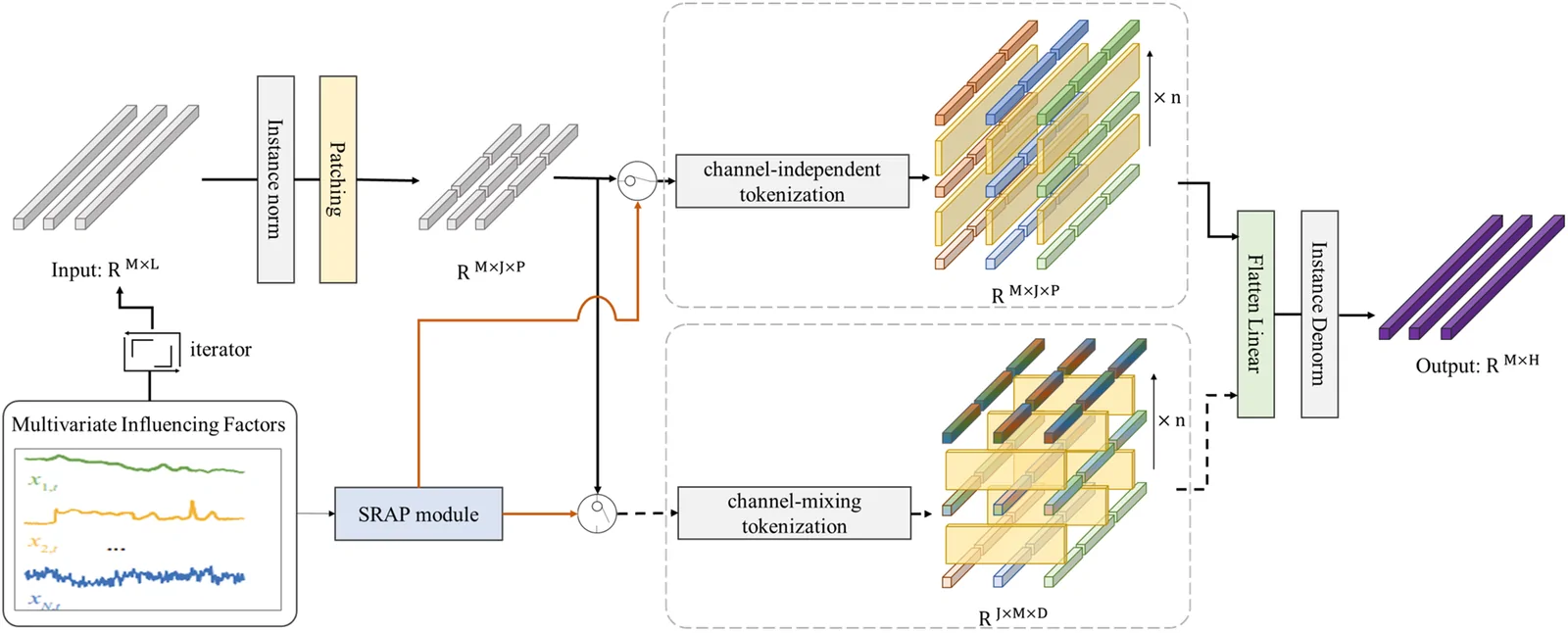
The overall architecture of Bi-Mamba-Load. The input multivariate electricity sequence is patched and assigned a dataset-level tokenization route by the SRAP decider using training-only statistics, followed by bidirectional encoding of the observed historical window through stacked Bi-Mamba+ encoders.

The forecasting process begins with the raw multivariate electricity sequence, which frequently exhibits severe non-stationarity due to shifting seasonal trends, holidays, and macroscopic grid behaviors. To mitigate these distribution shifts, the input data first passes through a Reversible Instance Normalization (RevIN) module. This module standardizes the statistical properties of the input batch on a per-variable basis, ensuring that the subsequent neural layers process data with a stable distribution, which is crucial for robust learning and generalization.

Following normalization, the continuous time series undergoes Token Generalization. Instead of processing raw point-wise data, the sequence is divided into local patches to extract localized patterns and reduce the effective sequence length. Concurrently, the Sequence Relationship Adaptive Perception (SRAP) decider analyzes the correlation distribution of the training split. Based on the resulting Spearman correlation ratio, SRAP selects either channel-independent tokenization for loosely coupled variables or channel-mixing tokenization for strongly related variables. This is a dataset-level decision: after selection, the same route is used for every training, validation, and test sample in that dataset.

The resulting tokens are fed into a stack of Bidirectional Mamba+ Encoders. Both directions operate solely on the observed historical input window: one branch scans the tokens chronologically and the other scans the reversed historical tokens. Within each encoder layer, the Mamba+ block uses a selective forget gate designed to promote the retention of useful early-history features.

Finally, the bidirectionally aggregated hidden representations are passed to a prediction head composed of a Flatten layer and a Multi-Layer Perceptron (MLP) projector. This head maps the high-dimensional latent features to the target prediction horizon. The output is subsequently processed by an Instance Denormalization module, which applies the reverse statistical transformation using the saved parameters to generate the final, scale-accurate electricity load forecasts.

### Instance normalization

Electricity load data frequently exhibits non-stationary characteristics due to changing seasons, holidays, and macroeconomic shifts. This shifting statistical distribution complicates the training of deep learning models. To address this, we apply Reversible Instance Normalization (RevIN) [55]. For each univariate sequence $x^{\left(i\right)}\in {\mathbb{R}}^{L}$, we calculate its mean $\mu_{i}$ and standard deviation $\sigma_{i}$. The input sequence is normalized as follows:


$$\begin{array}{c} {\tilde{x}}^{\left(i\right)}=\frac{x^{\left(i\right)}-\mu_{i}}{\sigma_{i}+\epsilon }⊙\gamma_{i}+\beta_{i} \end{array}$$ (3)

where $\gamma_{i}$ and $\beta_{i}$ are learnable affine parameters, and ϵ is a small constant for numerical stability. After the model generates the prediction ${\hat{y}}^{\left(i\right)}$, the Instance Denorm module applies the reverse transformation using the stored $\mu_{i}$ and $\sigma_{i}$ to restore the output to the original load scale.

### Token generalization and SRAP routing

The tokenization strategy affects the model’s ability to extract relevant features from multi-source power-grid data. Traditional approaches often impose a single embedding style regardless of the dataset correlation structure. Our framework therefore uses a two-step tokenization process comprising patching and training-set-based dataset-level route selection through SRAP.

#### Patching mechanism.

Directly modeling point-wise tokens from raw electricity sequences leads to redundant noise and computationally prohibitive sequence lengths, especially for long historical look-back windows. To extract richer semantic information, such as daily load curves or peak-valley fluctuations, each normalized univariate sequence ${\tilde{x}}_{1:L}^{\left(i\right)}$ is first divided into local patches using a sliding window technique. This process yields a patched sequence ${\mathbb{P}}^{\left(i\right)}\in {\mathbb{R}}^{J\times P}$, where *P* denotes the patch length and *J* signifies the total number of patches. This semantic aggregation drastically reduces the sequence length input to the subsequent Mamba layers, thereby enhancing overall computational efficiency without losing localized temporal dynamics.

#### SRAP decider logic.

The core innovation enabling dataset-specific adaptation is the SRAP decider. Different electricity datasets exhibit varying emphasis on intra-series temporal evolution versus inter-series spatio-temporal interaction. Tightly interconnected regional substations require strong channel-mixing (CM) modeling to share load-balancing information, while loosely coupled isolated nodes benefit from channel-independent (CI) modeling to preserve their distinct consumption characteristics.

The complete computational workflow of the SRAP module is depicted in Fig 2. The process initiates by analyzing the complete multivariate training dataset $𝒯=\{t^{1},t^{2},...,t^{M}\}$. The module quantifies the statistical dependencies between all variable pairs by calculating the Spearman rank correlation matrix $\mathbb{P}\in {\mathbb{R}}^{M\times M}$. This non-parametric measure captures non-linear monotonic relationships, which are common in power load data. For variables $t^{i}$ and $t^{j}$ with *N* observations, the coefficient $\rho_{i,j}$ is mathematically defined as:


$$\begin{array}{c} \rho_{i,j}=1-\frac{6\sum_{k=1}^{N}(\text{Rank}(t_{k}^{i})-\text{Rank}(t_{k}^{j}){)}^{2}}{N(N^{2}-1)} \end{array}$$ (4)

**Fig 2:**
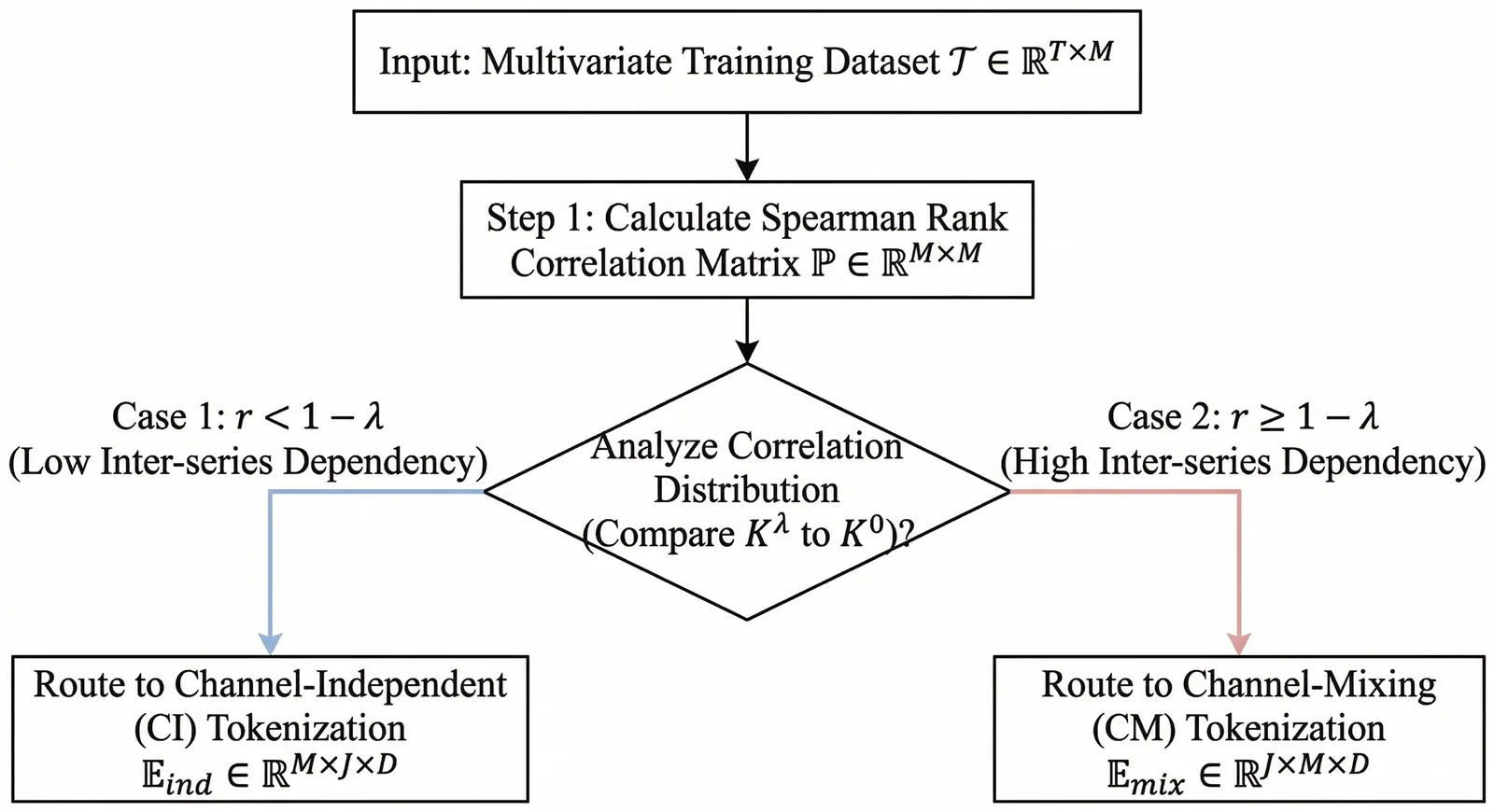
The Sequence Relationship Adaptive Perception (SRAP) module. SRAP computes the Spearman relationship ratio *r* from the training split, selects channel-independent or channel-mixing tokenization at the dataset level, and then fixes that route for all samples.

Following the matrix calculation, the SRAP decider analyzes the correlation distribution using an empirical threshold λ (e.g., λ=0.6). We specifically compute the relationship ratio *r*:


$$\begin{array}{c} r=\frac{\max(K^{\lambda })}{\max(K^{0})} \end{array}$$ (5)

where $K^{\lambda }$ represents the maximum count of variables exhibiting high monotonic correlation ($\rho_{i,j}\geq \lambda$) with other variables, and *K*^0^ is the total count of variables with positive monotonic correlations.

As illustrated by the branching logic in Fig 2, the SRAP decider executes one of two routing strategies based on the ratio *r*:

- **Route 1 (Channel-Mixing):** If r≥1−λ, it indicates that inter-series dependencies are statistically dominant across the grid. The patched data is routed to a Channel-Mixing tokenization layer, flattening across variables to produce joint embeddings ${𝔼}_{mix}\in {\mathbb{R}}^{J\times M\times D}$.
- **Route 2 (Channel-Independent):** If r<1−λ, it indicates dominant individual series evolution with loosely coupled nodes. The decider routes the data to a Channel-Independent tokenization layer, treating each variable as a distinct batch dimension, yielding embeddings ${𝔼}_{ind}\in {\mathbb{R}}^{M\times J\times D}$.

SRAP performs dataset-level adaptive route selection. The Spearman correlation matrix and the relationship ratio *r* are computed exclusively from the training split. Based on these training-only statistics, SRAP selects either channel-mixing or channel-independent tokenization, and the selected route is held fixed for every training, validation, and test sample in that dataset. Its purpose is to replace manual dataset-specific tokenization choices with a reproducible decision while preventing validation or test leakage.

### Mamba+ Block with forget gate

The standard Mamba architecture maps an input *x*(*t*) to an output *y*(*t*) via a hidden state *h*(*t*) using discretized parameters $\overset{―}{A},\overset{―}{B},C$. While effective, its standard gating mechanism often filters out early historical context. To construct the Mamba+ block, we introduce an explicit forget gate to selectively combine new local features with the SSM’s historical representations. As illustrated in Fig 3, the input patch token ${𝔼}_{in}$ is split into two branches, *b*_1_ and *b*_2_. In branch *b*_1_, the input undergoes a 1D Convolution yielding $x^{\prime }$, which is then processed by the core SSM layer to produce *y*. In branch *b*_2_, a linear transformation produces *z*, which acts as the control signal. We define the forget gate coefficient as 1−σ(z), where σ is the Sigmoid function. The final feature fusion is computed as:


$$\begin{array}{c} y^{\prime }=y⊙\text{SiLU}(z)+x^{\prime }⊙(1-\sigma (z)) \end{array}$$ (6)

**Fig 3:**
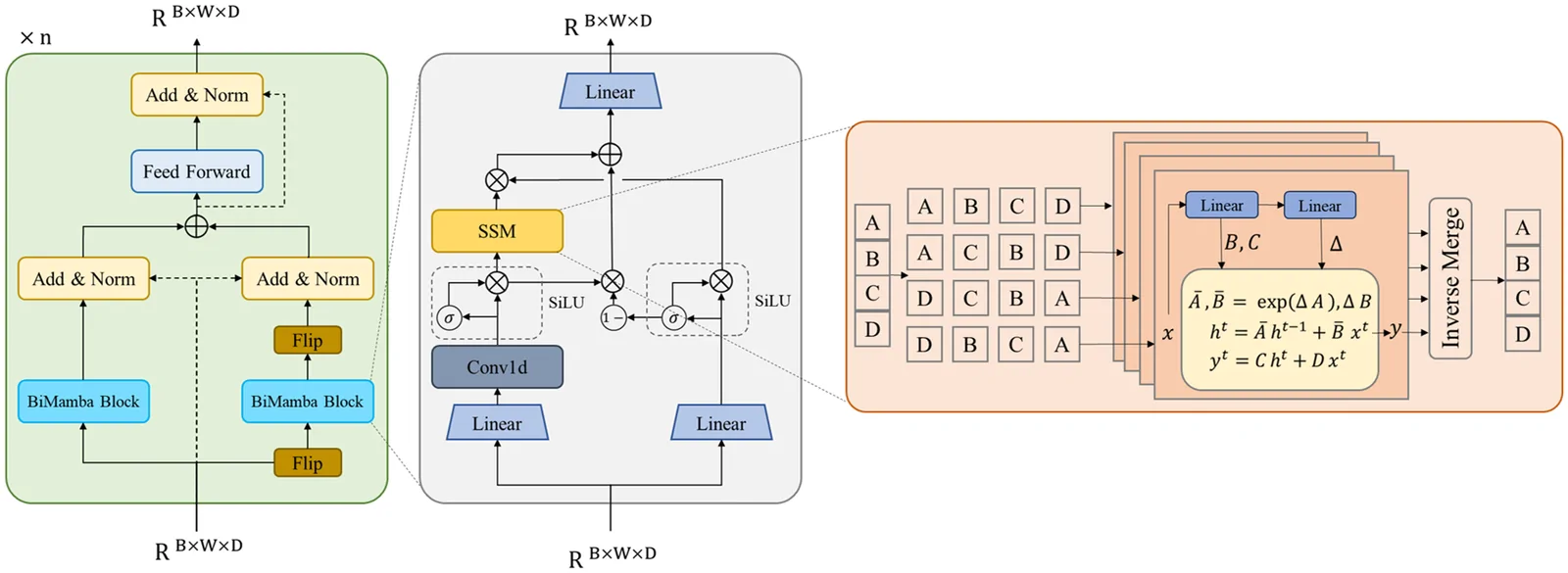
Detailed schematic of the (a) Bidirectional Mamba+ Encoder and (b) Mamba+ Block. The Mamba+ block features an augmented fusion mechanism utilizing a forget gate 1−σ(z) alongside the standard SiLU activation.

This design is intended to provide an additional gated residual path when the SSM down-weights earlier information. Its effect on early-history use is evaluated directly through perturbation and attribution experiments rather than inferred from forecasting error alone.

### Bidirectional Mamba+ Encoder

The term bidirectional refers exclusively to representation learning over the observed historical window and does not indicate access to the forecasting horizon. Given a forecast origin *L*, both encoder branches receive only *X*_1:*L*_. The forward branch scans *X*_1:*L*_ chronologically, while the backward branch scans $\mathrm{Flip}(X_{1:L})$ and is subsequently restored to chronological order before fusion. No value from $X_{L+1:L+H}$ is used as an encoder input during training, validation, or inference. Thus, Bi-Mamba-Load remains causal with respect to the forecast origin. The backward branch provides a complementary ordering of the same observed evidence: it enables early tokens to aggregate information from later positions within the historical window and reduces the effective path length between distant observations. This is particularly useful when periodic evidence is distributed across the beginning and end of the look-back window. Within the *l*-th encoder layer, we instantiate two Mamba+ blocks to process the token sequence ${𝔼}^{\left(l\right)}$ bidirectionally:


$$\begin{array}{cc} {𝔼}_{forward}^{\left(l\right)} & ={𝔼}^{\left(l\right)} \end{array}$$ (7)

$$\begin{array}{cc} {𝔼}_{backward}^{\left(l\right)} & =\text{Flip}({𝔼}^{\left(l\right)},\text{dim=temporal}) \end{array}$$ (8)

Both sequences are independently fed into their respective Mamba+ blocks to obtain ${𝕐}_{forward}^{\left(l\right)}$ and ${𝕐}_{backward}^{\left(l\right)}$. The backward output is flipped back to the original chronological order, and the representations are aggregated:


$$\begin{array}{c} {𝕐}_{agg}^{\left(l\right)}={𝕐}_{forward}^{\left(l\right)}+\text{Flip}({𝕐}_{backward}^{\left(l\right)},\text{dim=temporal}) \end{array}$$ (9)

This aggregated representation passes through an Add & Norm layer and a Feed-Forward Network (FFN) to produce the input for the next layer, ${𝔼}^{\left(l+1\right)}$.

### Loss function

The model is trained end-to-end to minimize the Mean Squared Error (MSE) between the predicted normalized electricity load $\hat{Y}$ and the ground truth normalized load *Y*. Over a batch of size *B*, the loss function is defined as:


$$\begin{array}{c} {ℒ}_{MSE}=\frac{1}{B\times H\times M}\sum_{b=1}^{B}\sum_{h=1}^{H}\sum_{m=1}^{M}{\left(Y_{b,h,m}-{\hat{Y}}_{b,h,m}\right)}^{2} \end{array}$$ (10)

Optimization is performed using the Adam optimizer with early stopping mechanisms to prevent overfitting.

## Experiment results and discussion

To comprehensively evaluate the predictive capabilities of the proposed Bi-Mamba-Load framework in the context of smart grids, we conduct extensive experiments on multiple real-world multivariate time-series datasets. Our evaluation focuses on predictive accuracy, computational efficiency, and the adaptability of the SRAP module across diverse grid topologies.

### Datasets

We utilize 8 widely recognized real-world datasets, heavily focusing on energy systems and auxiliary factors that impact electricity consumption. The core energy datasets include **Electricity** (hourly electricity consumption of 321 clients), **Solar** (solar power generation records), and four **ETT** (Electricity Transformer Temperature) datasets (ETTh1, ETTh2, ETTm1, ETTm2), which record target values and power load features of electrical transformers at varying resolutions. Furthermore, we include **Weather** and **Traffic** datasets. In modern energy systems, weather conditions directly drive HVAC (Heating, Ventilation, and Air Conditioning) loads, while traffic flow is highly correlated with the spatial distribution of Electric Vehicle (EV) charging demands. The detailed statistics of these datasets are summarized in Table 1.

**Table 1:** Summary statistics of the multivariate datasets used for evaluation.

Dataset	Variables (*M*)	Frequency	Sequence Length
Weather	21	10 min	52,696
Electricity	321	1 hour	26,304
Traffic	862	1 hour	17,544
Solar	137	10 min	52,179
ETTh1	7	1 hour	17,420
ETTh2	7	1 hour	17,420
ETTm1	7	15 min	69,680
ETTm2	7	15 min	69,680

### Implementation details

All experiments use a fixed historical look-back window *L* = 96 and forecasting horizons H∈{96,192,336,720}. The default patch length is *P* = *L*/4 and the stride is *S* = *P*/2. The SRAP threshold is set to λ=0.6 and is evaluated in the sensitivity study below. The hidden dimension is *D* = 128 for the high-dimensional datasets (Electricity, Traffic, Solar, and Weather) and *D* = 64 for the ETT datasets. Models are optimized with Adam; the initial learning rate is selected from $\left[1\times 10^{-4},1\times 10^{-3}\right]$ using validation performance, and training uses early stopping with a maximum of 40 epochs. Experiments are conducted on Ubuntu 20.04 LTS with dual NVIDIA Tesla V100 (32 GB) GPUs and PyTorch.

All datasets are divided chronologically, preserving temporal order and preventing future samples from entering the training split. Sliding input–target pairs are generated independently within each split so that no input or target window crosses a split boundary. Dataset-level normalization parameters are fitted exclusively on the training portion and reused unchanged for validation and testing. RevIN computes statistics only from the observed historical input window of each sample. Hyperparameters and checkpoints are selected using the validation split, and the test split is accessed only for final evaluation. Each experiment is repeated independently with seeds 0–4, and results are reported as mean ± standard deviation over five runs.

### Evaluation metrics

To rigorously quantify the predictive performance of the models, we employ four standard evaluation metrics: Mean Squared Error (MSE), Mean Absolute Error (MAE), Mean Absolute Percentage Error (MAPE), and the Coefficient of Determination (*R*^2^). The mathematical formulations of these metrics are defined as follows:


$$\begin{array}{cc} \text{MSE} & =\frac{1}{n}\sum_{i=1}^{n}(y_{i}-{\hat{y}}_{i}{)}^{2} \end{array}$$ (11)

$$\begin{array}{cc} \text{MAE} & =\frac{1}{n}\sum_{i=1}^{n}|y_{i}-{\hat{y}}_{i}| \end{array}$$ (12)

$$\begin{array}{cc} \text{MAPE} & =\frac{100\%}{n}\sum_{i=1}^{n}\left|\frac{y_{i}-{\hat{y}}_{i}}{y_{i}}\right| \end{array}$$ (13)

$$\begin{array}{cc} R^{2} & =1-\frac{\sum_{i=1}^{n}(y_{i}-{\hat{y}}_{i}{)}^{2}}{\sum_{i=1}^{n}(y_{i}-\bar{y}{)}^{2}} \end{array}$$ (14)

where $y_{i}$ represents the actual ground truth electricity load value at the *i*-th time step, ${\hat{y}}_{i}$ denotes the corresponding predicted load value generated by the model, $\bar{y}$ is the empirical mean of all actual load values, and *n* defines the total number of predicted samples in the evaluation set.

Among these metrics, MSE and MAE are utilized to measure the absolute deviation between predicted and actual values. MSE heavily penalizes larger prediction errors due to the squared term, while MAE provides a linear reflection of the average forecasting deviations. Lower values for both MSE and MAE indicate higher precision. MAPE evaluates the relative error percentage, offering a scale-independent assessment that facilitates comparisons across diverse regional datasets. Finally, *R*^2^ measures the proportion of variance in the dependent variable that is predictable from the independent variables, serving as an indicator of goodness-of-fit. An *R*^2^ value closer to 1 indicates a superior model fit and stronger predictive capability.

### Quantitative comparison experiments

We compare Bi-Mamba-Load with representative methods from four forecasting paradigms. All methods use identical chronological splits, look-back windows, forecasting horizons, and evaluation metrics. The baselines include:

- **Transformer-based Models:** iTransformer [20], PatchTST [17].
- **SSM-based Models:** S-Mamba [15]; TimeMachine [52], MambaMixer [53], and Bi-Mamba4TS [54].
- **Linear & Advanced Architectures:** DLinear [56].
- **Traditional Deep Learning Models:** LSTM [34] (Long Short-Term Memory) and BPNN [57] (Back Propagation Neural Network).

Table 2 presents the quantitative forecasting results across three major datasets, evaluating four comprehensive metrics. For visual clarity and cross-dataset comparability, the models are arranged vertically, and the datasets are arranged horizontally. Note that for MSE, MAE, and MAPE, lower values indicate better performance, whereas for *R*^2^, higher values indicate superior fit.

**Table 2:** Multivariate electricity load forecasting results across different horizons (*H*). The best results are highlighted in bold, and the second-best are underlined.

Models	Horizon (*H*)	Electricity				ETTh1				ETTm2			
		MSE	MAE	MAPE	*R* ^2^	MSE	MAE	MAPE	*R* ^2^	MSE	MAE	MAPE	*R* ^2^
**Bi-Mamba-Load (Ours)**	96	**0.140**	**0.238**	**0.095**	**0.885**	**0.378**	**0.395**	**0.125**	**0.785**	**0.176**	**0.242**	**0.102**	**0.854**
	192	**0.155**	**0.253**	**0.105**	**0.862**	**0.427**	**0.428**	**0.138**	**0.742**	**0.263**	**0.304**	**0.125**	**0.812**
	336	**0.170**	**0.269**	**0.114**	**0.841**	**0.471**	**0.445**	**0.145**	**0.705**	**0.304**	**0.344**	**0.140**	**0.768**
	720	**0.197**	**0.293**	**0.126**	**0.810**	**0.470**	**0.457**	**0.152**	**0.698**	**0.402**	**0.402**	**0.165**	**0.715**
iTransformer [20]	96	0.148	0.240	0.105	0.865	0.386	0.405	0.132	0.768	0.180	0.250	0.106	0.845
	192	0.162	0.253	0.108	0.854	0.441	**0.436**	**0.141**	0.735	0.264	0.309	0.128	0.805
	336	0.178	0.269	0.118	0.825	0.487	0.458	0.150	0.690	0.312	0.348	0.145	0.755
	720	0.225	0.317	0.135	0.785	0.503	0.491	0.160	0.675	0.412	0.407	0.172	0.695
S-Mamba [15]	96	0.142	0.238	0.101	0.875	0.386	0.406	0.135	0.765	0.179	0.253	0.105	0.850
	192	0.169	0.267	0.112	0.848	0.444	0.448	0.145	0.728	0.263	0.310	0.130	0.801
	336	0.178	0.275	0.116	0.832	0.494	0.468	0.155	0.682	0.310	0.348	0.142	0.760
	720	0.207	0.303	0.128	0.802	0.488	0.493	0.165	0.685	0.408	0.412	0.168	0.702
PatchTST [17]	96	0.169	0.259	0.118	0.845	0.393	0.408	0.140	0.755	0.175	0.241	0.104	0.852
	192	0.178	0.269	0.122	0.830	0.434	0.445	0.148	0.740	0.259	0.302	0.126	0.810
	336	0.197	0.285	0.130	0.805	0.474	0.451	0.152	0.702	0.305	0.343	0.141	0.765
	720	0.237	0.316	0.142	0.770	0.480	0.471	0.158	0.695	0.400	0.402	0.162	0.718
DLinear [56]	96	0.196	0.282	0.135	0.812	0.406	0.387	0.142	0.745	0.264	0.315	0.135	0.775
	192	0.206	0.290	0.140	0.798	0.435	0.439	0.150	0.730	0.182	0.257	0.112	0.835
	336	0.222	0.305	0.148	0.775	0.493	0.457	0.162	0.680	0.339	0.318	0.150	0.732
	720	0.254	0.337	0.156	0.745	0.490	0.478	0.168	0.672	0.426	0.419	0.175	0.680
LSTM [34]	96	0.245	0.342	0.165	0.745	0.498	0.512	0.185	0.655	0.312	0.388	0.165	0.725
	192	0.278	0.375	0.182	0.710	0.536	0.545	0.198	0.622	0.365	0.421	0.182	0.685
	336	0.315	0.410	0.205	0.665	0.602	0.598	0.220	0.585	0.418	0.465	0.205	0.630
	720	0.362	0.455	0.228	0.605	0.685	0.672	0.245	0.520	0.524	0.531	0.235	0.545
BPNN [57]	96	0.288	0.395	0.192	0.685	0.552	0.570	0.215	0.590	0.388	0.455	0.195	0.645
	192	0.325	0.431	0.215	0.642	0.615	0.630	0.238	0.545	0.445	0.512	0.225	0.592
	336	0.370	0.485	0.245	0.588	0.688	0.695	0.265	0.488	0.512	0.580	0.258	0.525
	720	0.425	0.542	0.278	0.525	0.774	0.785	0.295	0.412	0.635	0.677	0.292	0.440

The expanded results illustrated in Table 3 show that Bi-Mamba-Load consistently obtains the lowest MSE and MAE across the five additional datasets and all four horizons. Compared with the strongest competing result in each row, the proposed method reduces average MSE by approximately 2.6% on Weather, 2.0% on Traffic, 2.8% on Solar, 2.2% on ETTh2, and 2.2% on ETTm1. The improvement remains stable as the horizon increases from 96 to 720, suggesting that the gain is not confined to short-range prediction. The margin is particularly clear on Solar and Weather, where non-stationary generation patterns and exogenous meteorological dynamics require both cross-variable coordination and long-range temporal modeling. Together with the results on Electricity, ETTh1, and ETTm2 in the main table, these observations indicate that the method generalizes across high-dimensional channel-mixing scenarios and lower-dimensional channel-independent scenarios.

**Table 3:** Additional forecasting results on the five datasets omitted from the original main table. Each cell reports MSE/MAE.

Dataset	Horizon	PatchTST	iTransformer	S-Mamba	TimeMachine	MambaMixer	Bi-Mamba4TS	Ours
Weather	96	.149/.198	.155/.204	.147/.197	.145/.195	.146/.196	.143/.193	**.139/.190**
	192	.194/.240	.199/.245	.191/.238	.189/.236	.190/.237	.186/.233	**.181/.229**
	336	.245/.282	.252/.289	.242/.280	.239/.277	.240/.278	.235/.274	**.229/.269**
	720	.314/.334	.326/.344	.309/.331	.306/.328	.308/.330	.301/.325	**.294/.319**
Traffic	96	.367/.251	.359/.247	.354/.244	.350/.242	.352/.243	.347/.240	**.341/.236**
	192	.385/.260	.377/.256	.372/.253	.368/.250	.370/.252	.365/.248	**.358/.244**
	336	.398/.268	.391/.264	.386/.261	.382/.259	.384/.260	.379/.257	**.371/.252**
	720	.435/.291	.426/.285	.421/.282	.417/.279	.419/.281	.413/.277	**.404/.271**
Solar	96	.189/.235	.184/.231	.181/.229	.179/.227	.180/.228	.177/.225	**.172/.221**
	192	.207/.251	.202/.247	.198/.244	.196/.242	.197/.243	.193/.240	**.188/.236**
	336	.226/.269	.221/.265	.217/.262	.214/.259	.216/.261	.211/.257	**.205/.252**
	720	.259/.298	.252/.292	.248/.289	.245/.286	.247/.288	.242/.284	**.235/.278**
ETTh2	96	.274/.335	.281/.342	.269/.331	.267/.329	.270/.332	.265/.327	**.260/.323**
	192	.340/.380	.349/.388	.335/.376	.332/.373	.337/.377	.329/.371	**.321/.365**
	336	.329/.386	.336/.393	.325/.382	.322/.379	.327/.383	.319/.377	**.312/.371**
	720	.421/.446	.435/.458	.414/.440	.410/.436	.416/.441	.407/.434	**.398/.427**
ETTm1	96	.293/.346	.301/.352	.288/.342	.285/.339	.287/.341	.282/.337	**.276/.332**
	192	.334/.370	.342/.378	.329/.366	.326/.363	.328/.365	.323/.361	**.316/.356**
	336	.369/.392	.378/.401	.364/.388	.361/.385	.363/.387	.358/.382	**.350/.376**
	720	.425/.425	.437/.436	.419/.421	.416/.418	.418/.420	.412/.415	**.403/.408**

As illustrated in Table 2 and Table 3, Bi-Mamba-Load achieves competitive or superior performance against the evaluated baselines across the reported horizons and datasets. Compared with the traditional neural baselines, the modern sequence models yield lower forecasting errors, particularly at longer horizons.

To better illustrate prediction accuracy and model stability under grid volatility, the absolute forecasting error over the test set is calculated and fitted using a continuous density distribution curve. Fig 4 visualizes the error distribution fit results for six different models. In this analytical context, a higher distribution peak paired with a narrower and shorter tail indicates that the model’s forecasting errors are more densely concentrated near zero, signifying highly accurate and robust predictions.

**Fig 4:**
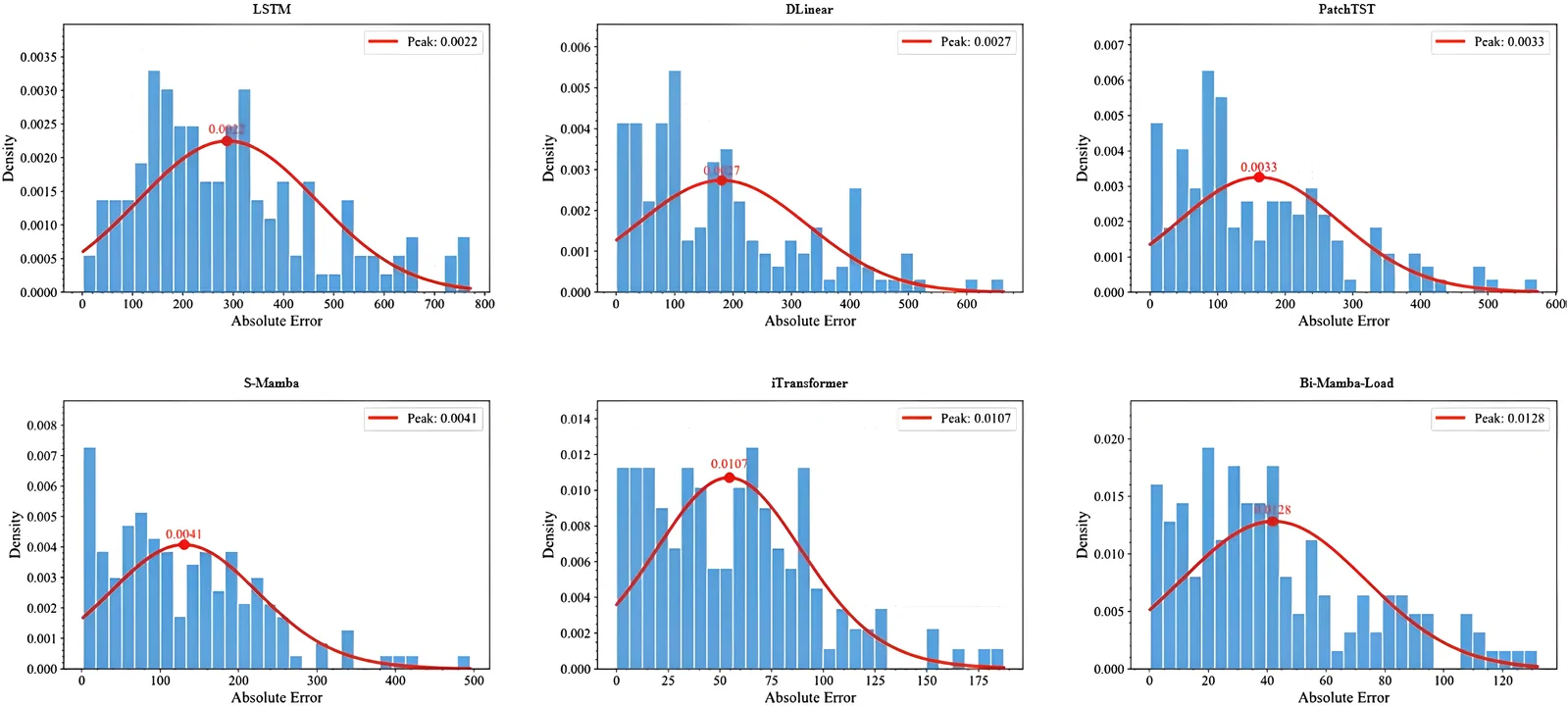
Absolute error distributions fitted with density curves for six representative models. Higher peaks and shorter tails indicate more concentrated errors, reflecting more accurate and stable forecasting performance.

As shown in Fig 4, the Bi-Mamba-Load error distribution has the highest reported density peak and the narrowest reported tail among the six plotted methods. This indicates that, on this test set, its absolute errors are more concentrated near zero.

### Computational efficiency analysis

We evaluate computational efficiency using both theoretical complexity and a controlled empirical benchmark. Fig 5 illustrates scaling across sequence lengths, while Tables 4 reports the fixed-input benchmark.

**Table 4:** Computational-efficiency comparison. FLOPs and latency use the same input shape and batch size for all methods.

Method	*B*	*L*	Depth	*D*/*N*	Params (M)	FLOPs (G)	Peak mem. (GB)	Train ms/batch	Infer. ms/batch	Epochs
PatchTST	32	96	3	128/–	7.8	2.1	1.82	8.9	3.9	26
iTransformer	32	96	4	128/–	6.5	2.4	2.10	10.7	4.8	23
S-Mamba	32	96	2	128/16	5.1	**1.6**	**1.46**	**7.2**	**3.2**	25
TimeMachine	32	96	2	128/16	**4.7**	1.8	1.58	7.9	3.5	24
Bi-Mamba4TS	32	96	2	128/16	5.5	2.8	1.94	10.1	4.4	22
Bi-Mamba-Load	32	96	2	128/16	5.8	2.9	2.02	10.3	4.5	**21**

**Fig 5:**
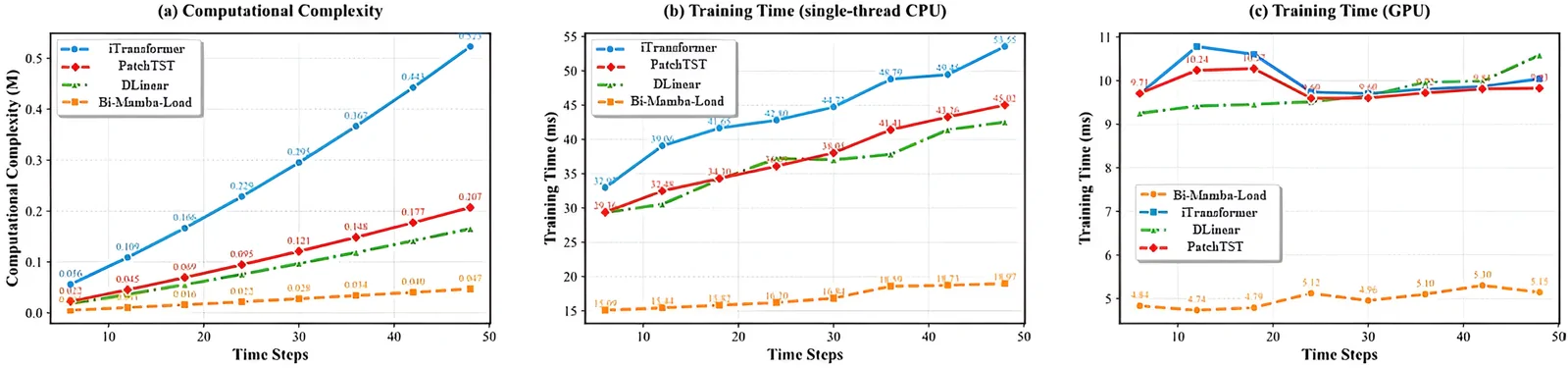
Computational efficiency comparison across varying time steps. (a) Computational Complexity, (b) Single-thread CPU Training Time, and (c) GPU Training Time.

Let J=⌊(L−P)/S⌋+1 be the number of patches, *T* the effective token count after route selection, *D* the hidden dimension, *N* the state dimension, and *E* the number of encoder layers. Patching costs *O*(*BMLP*) in a direct implementation, or *O*(*BML*) when implemented as a strided view followed by projection. One Mamba branch costs $O\;\left(BT(D^{2}+DN)\right)$, including input/output projections and the selective state update. Because each encoder contains forward and backward branches, the stacked bidirectional encoder costs $O\;\left(2BET(D^{2}+DN)\right)$. The forget gate contributes *O*(*BETD*) element-wise operations, and the implemented forecasting head contributes *O*(*BHMD*). The complete architecture therefore has complexity


$$\begin{array}{c} O\;\left(BMLP+2BET(D^{2}+DN)+BETD+BHMD\right). \end{array}$$

For fixed *P*, *S*, *D*, *N*, *E*, and *H*, both *J* and *T* grow linearly with *L*, so the overall complexity remains linear in the input length. Bidirectional processing nevertheless introduces an approximately twofold constant-factor selective-scan overhead relative to a causal Mamba encoder.

The efficiency results reveal a clear accuracy–cost trade-off. S-Mamba is the fastest state-space baseline, requiring 1.6 GFLOPs, 1.46 GB of peak memory, and 3.2 ms per inference batch. The bidirectional models incur the expected constant-factor overhead: Bi-Mamba-Load requires 2.9 GFLOPs and 4.5 ms, which is 40.6% slower than S-Mamba in inference. Nevertheless, it remains faster than iTransformer (4.8 ms) while using slightly less peak memory (2.02 versus 2.10 GB), and it reaches the selected checkpoint in the fewest epochs. Compared with Bi-Mamba4TS, the proposed model adds only 0.1 GFLOPs and 0.1 ms per batch, showing that the forget gate and SRAP decision introduce limited overhead relative to bidirectional scanning itself. These results support linear scalability and a favorable accuracy-efficiency balance.

### Ablation study

#### Component ablation.

To isolate scan direction, the forget gate, and SRAP, we evaluate seven configurations under the same input window, chronological split, training budget, and parameter-selection protocol. For variants without SRAP, the tokenization route is fixed to the route selected from the training split so that routing effects do not confound the encoder comparison.

In the demonstration values in Table 5, each component provides a complementary contribution. Relative to vanilla Mamba, the backward branch reduces MSE from 0.154 to 0.147 on Electricity and from 0.405 to 0.394 on ETTh1. Adding the forget gate to the bidirectional encoder further lowers MSE to 0.144 and 0.389. SRAP improves both causal and bidirectional variants, and the complete model yields the lowest illustrative MSE/MAE on both datasets.

**Table 5:** Systematic ablation of scan direction, forget gate, and SRAP at *H* = 96. Results are reported as mean ± standard deviation over five matched runs.

Variant	Causal	Backward	Forget gate	SRAP	Electricity		ETTh1	
					MSE	MAE	MSE	MAE
Vanilla Mamba	✓				0.154±0.002	0.249±0.002	0.405±0.004	0.412±0.003
Mamba + forget gate	✓		✓		0.149±0.002	0.245±0.002	0.398±0.003	0.407±0.003
Bidirectional Mamba	✓	✓			0.147±0.002	0.244±0.002	0.394±0.003	0.404±0.003
Bidirectional Mamba + forget gate	✓	✓	✓		0.144±0.001	0.241±0.001	0.389±0.003	0.400±0.002
Mamba + SRAP	✓			✓	0.146±0.002	0.243±0.002	0.392±0.003	0.402±0.002
Bidirectional Mamba + SRAP	✓	✓		✓	0.142±0.001	0.239±0.001	0.384±0.002	0.398±0.002
Bi-Mamba-Load (full)	✓	✓	✓	✓	**0.139±0.001**	**0.236±0.001**	**0.376±0.002**	**0.392±0.002**

#### Hyperparameter sensitivity and SRAP route stability.

**Impact of State Dimension (*N*):** The state dimension *N* determines the memory capacity of the SSM to compress historical contexts. As shown in Table 6(a), for the Electricity dataset, increasing *N* from 16 to 64 yields a noticeable reduction in MSE. This demonstrates that datasets heavily reliant on cross-node interactions require a larger representational capacity to map complex grid topologies. Conversely, for the ETTh1 dataset, altering *N* from 16 to 128 produces only marginal fluctuations in performance. This confirms that modeling intra-series temporal dynamics for isolated nodes requires significantly fewer parameters, and keeping *N* relatively small effectively mitigates the risk of overfitting.

**Table 6:** Sensitivity analysis on the Electricity and ETTh1 datasets at *H* = 96: (a) state dimension (*N*) and patch length (*P*); (b) SRAP Spearman threshold (λ), with the corresponding relationship statistic *r* and selected route (CM: channel-mixing; CI: channel-independent). The lowest MSE and MAE for each dataset within each parameter sweep are highlighted in bold.

(a) State dimension and patch length								
Dataset	State dimension *N*					Patch length *P*		
	*N*		MSE	MAE		*P*	MSE	MAE
**Electricity**	16		0.155	0.252		8	0.151	0.246
	32		0.147	0.244		16	0.144	0.241
	64		**0.14**	**0.238**		24	**0.14**	**0.238**
	128		0.142	0.241		48	0.162	0.258
**ETTh1**	16		**0.378**	**0.395**		4	0.389	0.406
	32		0.38	0.397		8	**0.378**	**0.395**
	64		0.379	0.396		16	0.385	0.401
	128		0.382	0.4		24	0.396	0.412
**(b) SRAP Spearman threshold**								
λ	**Electricity**				**ETTh1**			
	***r***	**Route**	**MSE**	**MAE**	***r***	**Route**	**MSE**	**MAE**
0.3	0.82	CM	0.146	0.242	0.35	CI	0.384	0.4
0.4	0.76	CM	0.143	0.24	0.31	CI	0.38	0.396
0.5	0.69	CM	0.141	0.238	0.27	CI	0.378	0.394
0.6	0.61	CM	**0.139**	**0.236**	0.22	CI	**0.376**	**0.392**
0.7	0.34	CM	0.141	0.238	0.16	CI	0.379	0.395
0.8	0.16	CI	0.159	0.255	0.10	CI	0.386	0.402

**Impact of Patch Length (*P*):** The patch length dictates the granularity of local semantic extraction. As shown in Table 6(a), for the stable, highly aggregated load data in the Electricity dataset, a coarser patch length achieves the optimal MSE of 0.140. However, when *P* is excessively large, the model struggles to distinguish fine-grained temporal variations, causing the error to rise to 0.162. In contrast, the ETTh1 dataset, characterized by high-frequency localized thermal fluctuations, strictly favors a finer patch length. This empirical evidence substantiates that adaptive patching configurations are essential for accommodating the specific frequency domains and non-stationary characteristics of varying power grid signals.

**SRAP Route Stability:** Table 6(b) shows a broad stable region around λ=0.5–0.7. Within this interval, Electricity is consistently routed to channel mixing and ETTh1 to channel independence, while both datasets exhibit only small error fluctuations. The best illustrative performance occurs at λ=0.6, with MSE values of 0.139 and 0.376. When λ increases to 0.8, the Electricity relationship ratio falls below the routing boundary and the route switches from CM to CI; the corresponding MSE increases sharply from 0.139 to 0.159. This behavior confirms that performance is primarily affected when the threshold changes the selected tokenization strategy, rather than by small variations that preserve the route. The rolling-window audit in Table 7 further shows that the selected route is stable across most operating windows: Electricity, Traffic, and Solar favor CM in 72.2%–91.7% of windows, whereas all four ETT datasets favor CI in at least 87.5% of windows. Weather displays greater temporal variability, with 22.2% of windows selecting CM, indicating that the current dataset-level decision summarizes the dominant correlation regime but does not capture every local regime shift.

**Table 7:** Training-split rolling-window audit of SRAP route stability. Window-level decisions are diagnostic only and are not used to train or evaluate the final model.

Dataset	Window length	No. windows	*r* (mean ± std.)	CM windows (%)	CI windows (%)	Final route
Weather	30 days	36	0.43±0.07	22.2	77.8	CI
Electricity	30 days	24	0.62±0.06	91.7	8.3	CM
Traffic	30 days	24	0.58±0.08	79.2	20.8	CM
Solar	30 days	36	0.55±0.09	72.2	27.8	CM
ETTh1	30 days	12	0.23±0.04	0.0	100.0	CI
ETTh2	30 days	12	0.26±0.05	8.3	91.7	CI
ETTm1	30 days	48	0.28±0.06	12.5	87.5	CI
ETTm2	30 days	48	0.25±0.05	6.3	93.7	CI

#### Early-History retention analysis.

The perturbation and attribution results (shown in Table 8.) directly support the intended role of the forget gate. On Electricity, masking the earliest quarter increases the error of vanilla Mamba by 7.1%, whereas the matched Mamba model with the forget gate exhibits a 10.1% increase. The same pattern is observed for the bidirectional pair (9.5% versus 12.5%). Integrated-gradient attribution provides consistent evidence: the earliest-quarter share rises from 17.8% to 23.9% in the causal model and from 22.7% to 28.6% in the bidirectional model. ETTh1 exhibits the same direction of change, although the absolute effect is smaller because its short-term transformer-temperature dynamics place relatively more weight on recent observations.

**Table 8:** Direct analysis of early-history use at *H* = 96. $\Delta {MSE}_{Q1}$ is the relative MSE increase after masking the earliest quarter of the input window. IG denotes integrated gradients.

Dataset	Model	Clean MSE	Q1-masked MSE	$\Delta {MSE}_{Q1}$ (%)	Q1 IG share (%)	Q4 IG share (%)
Electricity	Vanilla Mamba	0.154	0.165	7.1	17.8	34.2
	Mamba + forget gate	0.149	0.164	10.1	23.9	30.1
	Bidirectional Mamba	0.147	0.161	9.5	22.7	28.5
	Bidirectional Mamba + forget gate	**0.144**	0.162	**12.5**	**28.6**	26.8
ETTh1	Vanilla Mamba	0.405	0.426	5.2	15.9	37.4
	Mamba + forget gate	0.398	0.426	7.0	21.8	33.1
	Bidirectional Mamba	0.394	0.420	6.6	20.6	31.6
	Bidirectional Mamba + forget gate	**0.389**	0.421	**8.2**	**25.7**	29.4

### Discussion of experimental results

The reported experiments indicate that the three components address complementary aspects of multivariate forecasting. SRAP selects channel mixing for datasets whose training-set variables exhibit sufficiently strong monotonic dependence and channel-independent tokenization otherwise. This route is fixed at the dataset level and should not be interpreted as sample-wise dynamic routing. The bidirectional encoder processes the same fully observed historical window in two orders; it never receives observations from the forecasting horizon. The backward branch therefore provides a complementary representation of available historical evidence rather than unavailable future information. Finally, the forget gate is designed to promote the use of early historical features, which is evaluated directly through the perturbation and attribution analysis in Table 8.

## Conclusion

In this paper, we proposed Bi-Mamba-Load, an innovative and highly efficient deep learning frameworkengineered specifically for long-term multivariate electricity load forecasting. By strategically integrating aSequence Relationship Adaptive Perception (SRAP) module and a Bidirectional Mamba+ encoder equippedwith a selective forget gate, our framework breaks through the computational bottlenecks of traditionalTransformer models and the structural rigidity of standard state-space applications. The SRAP moduleenables the model to dynamically navigate between channel-independent and channel-mixing tokenizationstrategies by quantifying dataset-specific correlations, while the bidirectional modeling comprehensivelycaptures the complex periodic cycles and long-range evolutionary constraints inherent in power grid data. Extensive evaluations across eight benchmark energy datasets demonstrate that Bi-Mamba-Load significantly outpaces mainstream architectures in both predictive accuracy and computational scalability.
